# Amphibian life history in a temperate environment of the Mexican Plateau: dimorphism, phenology and trophic ecology of a hylid frog, *Hyla eximia* (=*Dryophytes eximius*)

**DOI:** 10.7717/peerj.5897

**Published:** 2018-11-08

**Authors:** Uriel Hernández-Salinas, Aurelio Ramírez-Bautista, Barry P. Stephenson, Raciel Cruz-Elizalde, Christian Berriozabal-Islas, Carlos Jesús Balderas-Valdivia

**Affiliations:** 1 Instituto Politécnico Nacional, Centro Interdisciplinario de Investigación para el Desarrollo Integral Regional (CIIDIR) Unidad Durango, Durango, Durango, Mexico; 2 Laboratorio de Ecología de Poblaciones, Centro de Investigaciones Biológicas, Instituto de Ciencias Básicas e Ingeniería, Universidad Autónoma del Estado de Hidalgo, Pachuca, Hidalgo, Mexico; 3 Department of Biology, Mercer University, Macon, GA, USA; 4 Dirección General de Divulgación de la Ciencia, Zona Cultural de Ciudad Universitaria, Universidad Nacional Autónoma de México, Mexico City, Mexico

**Keywords:** Amphibian, Growth rate, Diet, Reproduction

## Abstract

The study of demographic and life history aspects of an organism provides valuable information for its conservation. Here, we analyze the phenology of the Mountain Treefrog *Hyla eximia* (= *Dryophytes eximius*) in a temperate environment of the Mexican Plateau. Females were larger in snout-vent length and body mass than males. The peak period of activity occurred in the rainy season (May–September), with amplexus and egg deposition occurring between June and July, and larval development from July to August. A logistic model best explained observed male growth patterns, while the Von Bertalanffy model better described female growth. Notably, males grew faster than females, although females reached a larger overall body size. The diet of this species is made up of 10 prey categories. The index of diet importance indicated that males feed mainly on Coleoptera and Diptera, while females feed on Coleoptera, Diptera, Hemiptera, and Aranea. Both females and males showed a significant abundance of plant material in their stomachs, suggesting that *H. eximia* might exhibit highly specialized feeding behavior. Reproduction was seasonal, and both female and male reproductive cycles are synchronized with the rainy season. These natural history characteristics provide information to better understand their responses to environmental conditions.

## Introduction

The geographic distribution of amphibians is closely related to their ecological, morphological and physiological characteristics (Dayton & Fitzgerald, 2006), as well as environmental conditions (Guisan & Zimmermann, 2000). In anurans, local environmental factors, such as temperature, precipitation, or humidity, directly influence species life history (Wells, 2007). Adaptation by each anuran species gives rise to diverse life history characteristics (e.g., reproduction, foraging, growing, survival); and a diverse range of survival and reproductive strategies (Egea-Serrano, Oliva-Paterna & Torralva, 2005; Crump, 2015). For example, in tropical and subtropical environments, the reproductive period of many species of treefrogs (family Hylidae) occurs throughout the year due to constant humidity and temperature conditions (Duellman & Trueb, 1986; Da Silva et al., 2012). Conversely, in arid or semi-arid environments, reproduction in toads occurs only during the rainy season (Denver, Mirhadi & Phillips, 1998; Boeing, Griffis-Kyle & Jungles, 2014).

In temperate high-elevation environments, the reproductive behaviors of anurans are also related to the onset of rains and environmental temperature (Duellman & Trueb, 1986; Vitt & Caldwell, 2013; Crump, 2015). For example, the spadefoot toad *Spea hammondii*, which inhabits arid environments of western North America, amplexus and egg deposition are synchronized with the first rains of the year (Duellman & Trueb, 1986; Denver, Mirhadi & Phillips, 1998). Similarly, in hylids from mountainous regions of Mexico, such as *Hyla plicata* or *H. euphorbiacea*, reproductive activity (such as mating calls and amplexus) occurs with the arrival of the rains, and egg laying and larval development continue until the end of this season (Luría-Manzano & Gutiérrez-Mayén, 2014; Lemos-Espinal et al., 2016). This indicates that physiologically and morphologically these species are adapted to the environments they inhabit; however, depending on the quality and quantity of available resources, the growth rates (GRs) of organisms may differ between sexes or populations (Schoener, 1974; Ricklefs & Schluter, 1993; Pleguezuelos, 1997; Ramírez-Bautista, Hernández-Salinas & Zamora-Abrego, 2016). This pattern has been observed in anuran species from tropical environments (Haddad & Prado, 2005; Crump, 2015) and temperate montane habitats (Luría-Manzano & Gutiérrez-Mayén, 2014; Cruz-Ruiz et al., 2015; Lemos-Espinal et al., 2016).

Having complex life cycles, frogs show evidence of strong influence of environmental factors on their species phenology, as well as the development of larvae, juveniles, and adults (Haddad & Sawaya, 2000). In Mexico, hylids inhabiting high mountains occur mainly in the center of the country (Faivovich et al., 2005). However, very little is known regarding the phenology, ecology, or behavior of such species from these environments (Duellman, 2001; Cruz-Ruiz et al., 2015; Lemos-Espinal et al., 2016), limiting our understanding of their population dynamics. This study addresses that deficiency by examining the life history characteristics of the Mountain Treefrog *H. eximia*, in turn providing the basis for future hypothesis-based approaches. The specific goals of this study were the following: (i) to determine if there is sexual dimorphism in a study population of *H. eximia* from Hidalgo, Mexico, (ii) to characterize the reproductive period of this population, identified by the production of mating calls, amplexus, egg laying, and larval development, and (iii) to assess the feeding habits and GRs of males and females of *H. eximia*.

## Methods

### Study species

**Figure 1:**
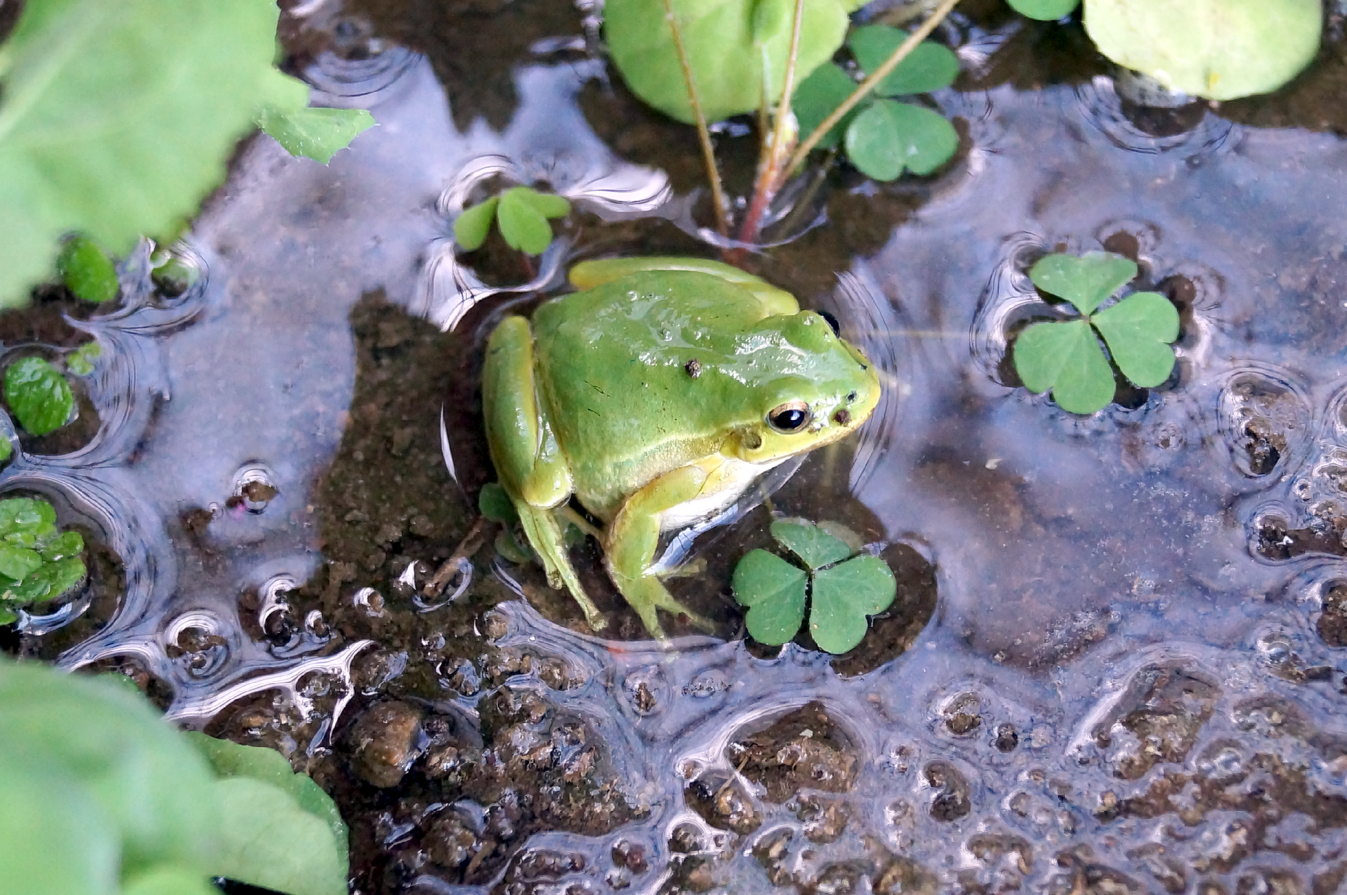
*Hyla eximia* from Rancho Santa Elena. Adult female *Hyla eximia* from Rancho Santa Elena in the municipality of Huasca de Ocampo, Hidalgo, Mexico. Photograph taken by Uriel Hernández-Salinas.

*Hyla eximia* (= *Dryophytes eximius*) is a small species (Fig. 1), with an adult snout-vent length (SVL) of 36.2 mm, endemic to temperate montane environments of central Mexico at elevations at 900–2,900 m asl (Duellman, 2001). Recent work argued for the placement of this taxon within the genus *Dryophytes* (Duellman, Marion & Hedges, 2016); here, we retain the use of the genus *Hyla* for the study species, while acknowledging that its taxonomic status at the generic level is not yet resolved.

### Study area and field work

This study was carried out from May 21 to September 24, 2013 at the Ecotourism Reserve Rancho Santa Elena, municipality of Huasca de Ocampo (20.1381 N,-98.5039W; WGS84), Hidalgo, Mexico. This site is located at an elevation of 2,400 m asl; mean annual precipitation is 543.3 mm, mean annual temperature is 14.8 °C (García, 1988), and the dominant vegetation type is pine forest (INEGI, 1992).

Field work consisted of five sampling periods of 3 days each during the rainy season (May–September). Sampling was conducted from 19:00 to 23:00 h coinciding with activity of adult individuals, within a transect 500 m long by 10 m wide where frogs were collected. To track frogs during the breeding season, we used the capture-mark-recapture method, marking subjects by toe-clipping (Campbell et al., 2009). In this study, a total of 128 adult individuals were captured (113 males and 15 females); of these, 30 males and 13 females were recaptured at least once, for a total of 62 recapture events overall. Immediately following capture, the body temperature of each frog, and microhabitat temperatures of capture sites, were collected with a quick reading Miller & Weber mercury cloacal thermometer (scale 0–50 °C). Additional morphological (SVL, body mass) and categorical data (sex, microhabitat, collecting time) were also collected in the field; afterward, marked frogs were released at their sites of capture.

During these same sampling periods, we also collected 15 adult males and 15 adult females in order to describe the reproductive condition of males (size and volume of testis) and females (size and weight of clutch), as well as feeding habits. Frogs were humanely sacrificed with an overdose of pentobarbital (0.1 ml) and subsequently fixed with 10% formalin (Casas-Andreu, Valenzuela-López & Ramírez-Bautista, 1991). Field work was conducted according to the Guidelines for the Care and Use of Lower Vertebrates (2004), and the national Mexican laws CT-CERN-001-91 (Diario Oficial de la Federación, 1991) and NOM-PA-CRN-001/93 (Diario Oficial de la Federación, 1993). Specimens were collected under scientific permit SGPA/DGVS/01902/11 provided by SEMARNAT. All preserved specimens were deposited in the amphibian and reptile collections of the Centro de Investigaciones Biológicas of the Universidad Autónoma del Estado de Hidalgo.

### Data analysis

To characterize sexual dimorphism, we measured the following characteristics: SVL (± 0.1 mm), head length (HL; ± 0.01 mm), head width (HW; ± 0.01 mm), arm length (AL; ± 0.01 mm), forearm length (FL; ± 0.01 mm), femur length (FEL; ± 0.01 mm), and tibia length (TL; ± 0.01 mm). All distance measures were taken with a digital caliper, and body mass was measured with a Pesola balance (5 and 10 g). We used averages of SVL and other morphological variables (HL, HW, AL, FL, FEL, and TL) of adults to calculate the sexual size dimorphism index (SSDi; Lovich & Gibbons, 1992) according to the equation: SSDi = (SVL of the larger sex/SVL of the smaller sex)–1. This value is positive if females were larger, and negative if males were larger (Lovich & Gibbons, 1992). For data on population phenology of *H. eximia*, we defined the period of reproductive activity (day and month) to be the time from the start to the end of calling in males. This period included observations of amplexus, egg deposition, presence of larval stages, and emergence of juveniles (Paton, Stevens & Longo, 2000). To assess reproductive condition, preserved frogs were dissected, and eggs or testes examined. For females, we recorded clutch size and mass (± 0.0001 g; Adam digital balance). To assess clutch sizes, eggs were placed in a petri dish and a stereoscopic microscope used to count the eggs (Hernández-Austria, Lara-Tufiño & Ramírez-Bautista, 2015). For males, we recorded testes mass (± 0.0001 g) and volume. Testis volume was determined using the formula of an ellipsoid, *V* = (4/3) π *a*^2^*b*, where *a* is half the shortest diameter and *b* is half the longest diameter (Dunham, 1978).

To evaluate the diet of females and males, stomach contents were removed and poured into a petri dish lined with millimeter-ruled graph paper. All insects (complete and semi-digested) found in each stomach were identified to order (Triplehorn & Johnson, 2005). Representatives of Hymenoptera were also divided into two groups, formicids and non-formicids (Luría-Manzano & Ramírez-Bautista, 2017). In addition, we counted the number of items of plant material (such as leaves, seeds, and fruits) as well as inorganic material (sand grains) found in each stomach. The length and width of each prey item were measured to obtain prey volume (Dunham, 1978; Luría-Manzano & Ramírez-Bautista, 2017). For each prey category, we calculated the abundance and frequency of occurrence (Luría-Manzano & Ramírez-Bautista, 2017).

Diet data were analyzed according to the importance index (*I*) using the formula of Biavati, Wiederhecker & Colli (2004), *I* = (N% + F% + V%)/3; where N% represents the numerical percentage (abundance), F% is the percentage of occurrence (frequency) and V% is the volumetric percentage. In addition, accounting for the types of prey consumed by each sex, the degree of food overlap was obtained through the Pianka index (O_*jk*_; Pianka, 1973). The formula is represented as: ${O_{jk}} = \sum\nolimits_{i = 1}^{\rm{n}} {{{\rm{P}}_{ij}}{{\rm{P}}_{ik}}/} \surd \sum\nolimits_{i = {\rm{ }}1}^{\rm{n}} {{\rm{P}}_{ij}^2\sum\nolimits_{i = 1}^{\rm{n}} {{\rm{P}}_{ij}^2} } $; where P_*ij*_ and P_*ik*_ are the proportions of abundance in the use of resource *i*, by the species *j* and *k*, which in this case are the sexes. This index ranges from 0 (when the resource use between the sexes is completely different) to 1 (when resource use is identical).

Capture and recapture data were used to assess the GRs of females and males of *H. eximia* (Ramírez-Bautista, 1995). GRs was determined as the difference between the second (SVL_2_) and first (SVL_1_) body lengths recorded, divided by the number of days between captures. Using non-linear regression techniques, we fit three different growth models (Von Bertalanffy, logistic-by-length, and logistic-by-weight; Dunham, 1978; Schoener & Schoener, 1978; Zamora-Abrego, Zúñiga-Vega & Ortega-León, 2012) to our data that relate GRs to body length (mean SVL between captures).

Growth rates were estimated for those individuals that were recaptured between 10 and 100 days after their previous captures, which prevented overestimating the growth values of each individual and sex (Zamora-Abrego, Zúñiga-Vega & Ortega-León, 2012). The choice of the best model was based on which represented the best fit for the GRs for each sex, chosen by the lowest values of the mean square residual and the highest coefficient of determination or correlation (*R*^2^: Dunham, 1978; Schoener & Schoener, 1978). We performed Spearman’s correlations (Zar, 2014) using environmental temperature and temperature of the microhabitat against the residuals of the GRs of both sexes to test whether one or both environmental factors influenced the growth of males and females. The residuals used in this correlation were obtained from the growth model (e.g., logistic-by-length) that showed the best fit for both sexes.

We used parametric tests when data met corresponding conditions for normality and homogeneity of variances (Levene’s test; Zar, 2014); otherwise, we used non-parametric tests. To test for differences in morphological variables between the sexes, we used non-parametric Mann–Whitney *U*-tests. We used regression analysis to test the relationship of HL, HW, AL, FL, FML, and TL with respect to SVL for each sex; We then used analysis of covariance (ANCOVA) to test for differences between sexes of the slopes of said regressions that represent the increase of the variables respect to SVL. We used Student’s *t*-tests to test for differences in body and microhabitat temperature between the sexes (Zar, 2014). Tests of analysis of variance (ANOVA) were used to assess differences of body mass and gonadal volume of males and females, as well as clutch size and egg mass across among months. All statistical analyzes were performed with Statistica version 7.0 (StatSoft, Inc., Tulsa, OK, USA), while the calculation of the Pianka index was performed using Ecological Methodology version 6.1.1 (Krebs, 2013). All means are reported ±standard error.

## Results

### Sexual dimorphism

Males were smaller than females in SVL (males: 29.9 ± 0.39 mm; range = 26.9–32.7 mm, *n* = 113; females: 33.5 ± 0.49 mm, range = 29.0–37.5 mm, *n* = 15; Mann–Whitney *U*-test = −4.656, *P* = 0.003) and body mass (males: 2.5 ± 0.39 g, range = 1.4–3.5 g; females: 3.4 ± 0.74 g, range = 2.0–4.4 g; Mann–Whitney *U*-test = −4.676, *P* = 0.001; Table 1). Similar patterns were recorded for other morphological variables using the index of sexual dimorphism, indicating that females showed larger morphological proportions (Table 1). The ANCOVAs indicated that increases in HL, HW, AL, FL, and FML were related to increases in SVL, and that these increases were significantly higher in females than in males (Tables 1 and 2). The slopes for TL were not different between sexes, although females exhibited higher overall TL than males (Table 2).

**Table 1:** Comparison of morphological variables between males and females of *Hyla eximia*.

Variables	Males	Females	SSDi	*t*	*P*
SVL	29.7 ± 1.56 (26.2–33.7)	32.5 ± 1.76 (29.9–35.1)	1.09	6.37	<0.0001
HL	9.93 ± 0.67 (8.8–11.6)	10.4 ± 0.95 (8.8–12.8)	1.04	2.52	0.013
HW	9.99 ± 0.68 (8.9–12.0)	10.9 ± 0.65 (9.9–12.1)	1.09	4.72	<0.0001
AL	6.63 ± 0.68 (4.9–7.9)	7.14 ± 0.41 (6.4–8.0)	1.07	2.82	0.005
FL	6.60 ± 0.65 (5.1–7.9)	7.02 ± 0.31 (6.5–7.7)	1.06	2.42	0.016
FML	15.34 ± 0.76 (14.0–17.4)	16.20 ± 1.16 (14.0–18.0)	1.05	3.84	0.0002
TL	15.59 ± 0.94 (13.5–17.9)	16.95 ± 1.13 (15.5–19.0)	1.08	5.1	<0.0001
BM	2.5 ± 0.39 (1.4–3.5)	3.4 ± 0.74 (2.0–4.4)		8.11	<0.0001

**Notes:**

Comparison of morphological variables between males (*n* = 113) and females (*n* = 15) of *Hyla eximia*.

Means for each variable reported ± standard error; values in parentheses show variable ranges. SVL, snout-vent length; HL, head length; HW, head width; AL, arm length; FL, forearm length; FML, femur length; TL, tibia length; BM, body mass; SSDi, Sexual size dimorphism index.

**Table 2:** Regression statistics and comparison of slopes regression of the relationships between morphological variables of males and females of *Hyla eximia*.

							ANCOVA (comparison of slopes)		
Variables	Sex	*R*^2^	F	d*f*	Slope	Intercept	*F*	d*f*	*P*
HL	F	0.69	29.53	1,14	0.45	−4.29	4.91	1,124	0.028
	M	0.33	56.37	1,112	0.25	2.44			
HW	F	0.68	28.31	1,14	0.33	0.95	0.11	1,124	0.021
	M	0.46	96.43	1,112	0.29	1.17			
AL	F	0.54	13.06	1,14	0.31	1.76	3.47	1,124	0.046
	M	0.51	119.62	1,112	0.16	−2.75			
FL	F	0.44	10.35	1,14	0.31	3.13	7.25	1,124	0.008
	M	0.57	149.35	1,112	0.12	−2.85			
FML	F	0.67	27.25	1,14	0.54	−1.41	8.68	1,124	0.003
	M	0.23	33.26	1,112	0.23	8.39			
TL	F	0.71	32.03	1,14	0.54	−0.71	0.82	1,124	0.366
	M	0.56	142.17	1,112	0.45	2.12			

**Notes:**

Regression statistics and comparison of slopes of regression of the relationship between morphological morphological variables of males (M) and females (F) of *Hyla eximia*. HL, head length; HW, head width; AL, arm length; FL, forearm length; FML, femur length; TL, tibia length.

### Phenology

**Figure 2:**
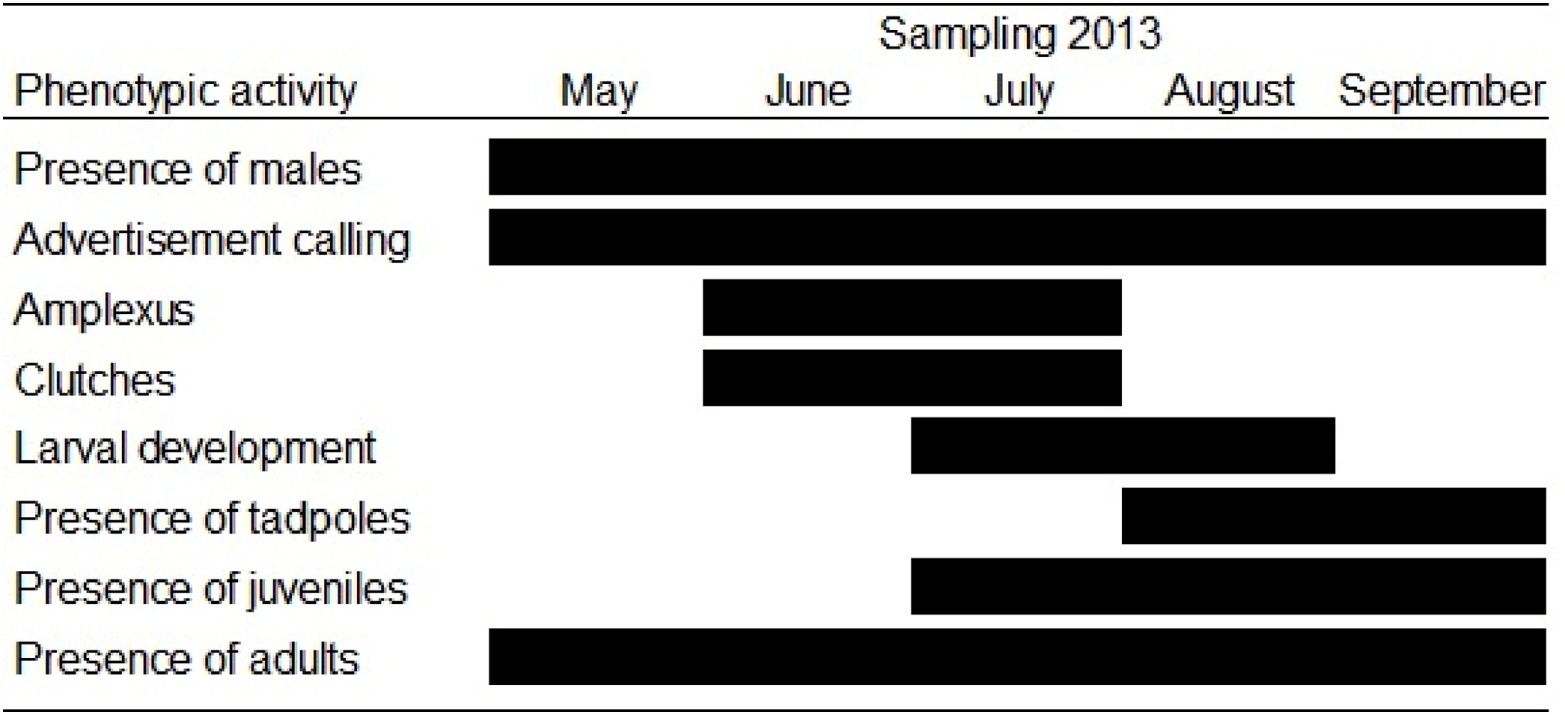
Phenological phases of *Hyla eximia*. Phenological phases observed during the sampling period (May–September 2013) for *Hyla eximia*.

From May to June (rainy season) we recorded adults producing mating calls and females depositing clutches, as well as juveniles and various larval stages (Fig. 2). Males called from May to September, whereas in June and July we observed pairs in amplexus in the vegetation at the edge of the water. The first egg clutches appeared in June and the last clutches were observed at the end of September (Fig. 2).

**Figure 3:**
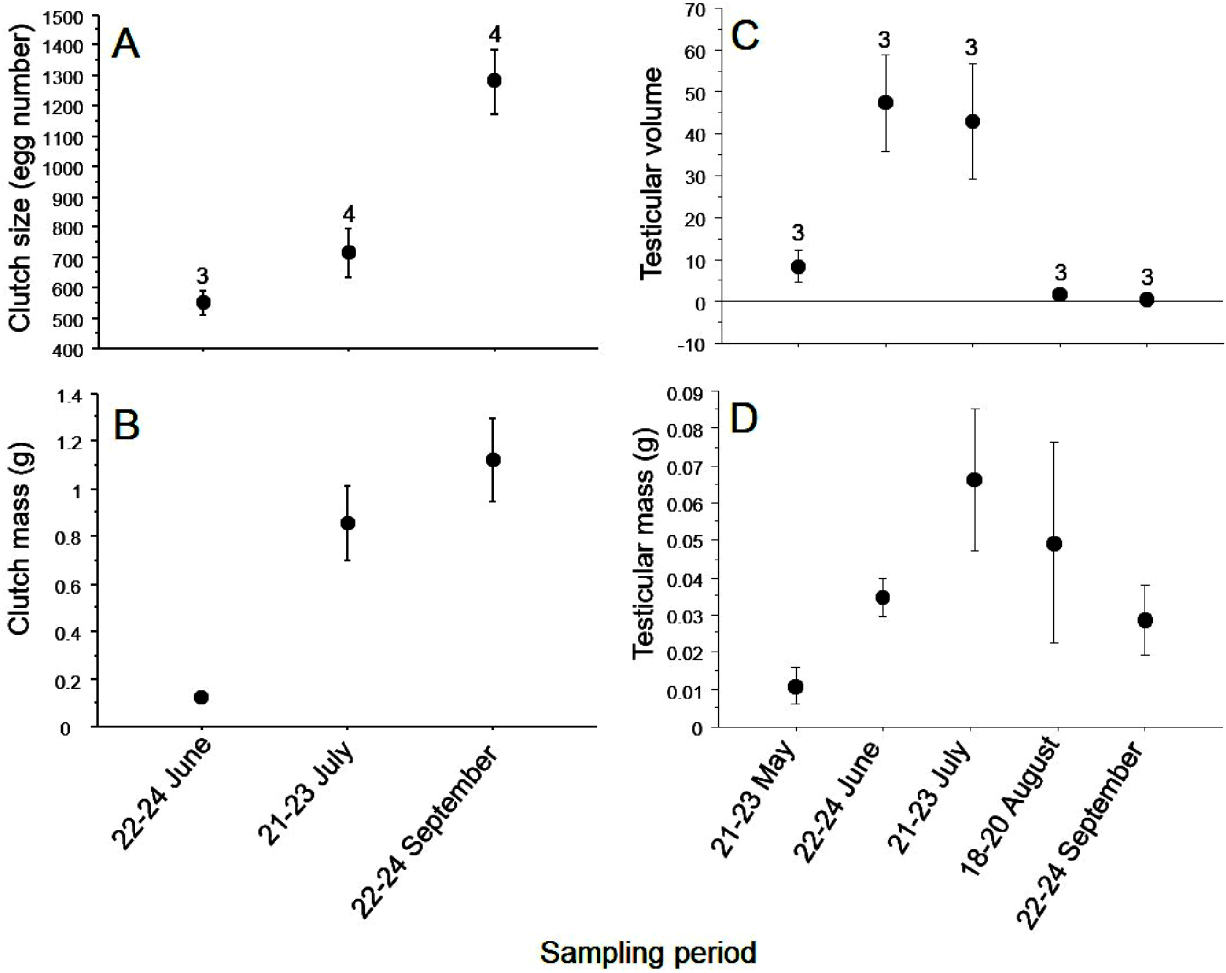
Monthly variation in size and weight of egg clutches of *Hyla eximia*. Monthly variation in size (A) and weight of egg clutches (B) in females, and in the volume (C) and weight (D) of testes of males in *Hyla eximia* (mean ± SE).

Larval development occurred between July and August, and during this time we observed a large number of tadpoles in different stages (Fig. 2). Mean clutch size was 851.1 ± 106.93 eggs (range = 508–1,476 eggs; *n* = 11), which differed among months (ANOVA, *F*_2,9_ = 14.54, *P* = 0.003). Mean clutch mass was 0.79 ± 0.14 g (range = 0.11–1.44 g; *n* = 11) and also differed among months (*F*_2,9_ = 6.41, *P* = 0.02; Figs. 3A and 3B). Testes mass was 0.039 ± 0.023 g (range = 0.06–0.12 g; *n* = 15), and did not differ among months (ANOVA, *F*_4,10_ = 1.93, *P* = 0.18). However, testes volume was 20.11 ± 6.33 mm (range = 0.038–0.098 mm^3^), and showed differences among months (*F*_4,10_ = 8.05, *P* = 0.003; Figs. 3C and 3D).

Adult females of *H. eximia* used microhabitats (perches) with higher air temperatures (19.5 ± 1.89 °C, range = 15.0–22.0 °C) than those used by males (17.3 ± 2.47 °C, range = 12.0–22.0 °C; *t* = 2.41, *P* = 0.04); however, no differences were found in body temperature between sexes (males: 19.9 ± 3.12 °C, range = 16.0–25.0 °C; females: 21.0 ± 2.55 °C, range = 17.0–25.0 °C; *t* = 1.11, *P* = 0.09).

### Diet

We analyzed the stomach contents of 30 adult frogs, and recorded a total of 134 prey, representing 10 categories distributed among Araneae and Insecta (Table 3), as well as plant matter. Males consumed 52 prey items, whereas females consumed 82 prey items, corresponding to seven and nine prey orders, respectively, without considering the plant matter (Table 3). Prey items that were most abundant in the diet of males were Coleoptera (26%) and Diptera (17%; Table 3), whereas for females the most abundant prey items were Coleoptera (25%), Diptera (20%), and Araneae (13.5%; Table 3). The prey items of highest volume consumed by males were coleopterans, and for females were hemipterans, coleopterans, and lepidopterans. According to the importance index, the most important prey for males were Coleoptera (*I* = 20.0) and Diptera (*I* = 6.2), and for females were Coleoptera (*I* = 25.5), Diptera (*I* = 21.2), Hemiptera (*I* = 17.2), and Araneae (*I* = 15.7; Table 3). The index of overlap was 67%, which indicates a similar diet between the sexes (O_*jk*_ males = 0.425; females = 0.498).

**Table 3:** Composition of the diet of adult males and females of *Hyla eximia* during the rain season at Rancho Santa Elena.

	Males			Females		
Prey category	IA	Ab	Vol	IA	Ab	Vol
Araneae	2	9	21.18	15.7	12	246.89
Coleoptera	20	13	1448.34	25.5	19	1630.30
Hymenoptera						
Formicidae	3.3	6	9.60	4.6	9	14.00
Non-Formicidae	1.5	4	109.93			
Lepidoptera	0.7	2	6.13	9.3	4	262.72
Orthoptera				6	5	29.29
Diptera	6.2	12	60.95	21.2	15	183.62
Hemiptera	2.6	6	79.45	17.2	6	2625.72
Odonata				7.5	8	67.10
Siphonoptera				3.2	4	8.30
Plant material	12	19	1083.93	9	17	18.83

### Growth

**Figure 4:**
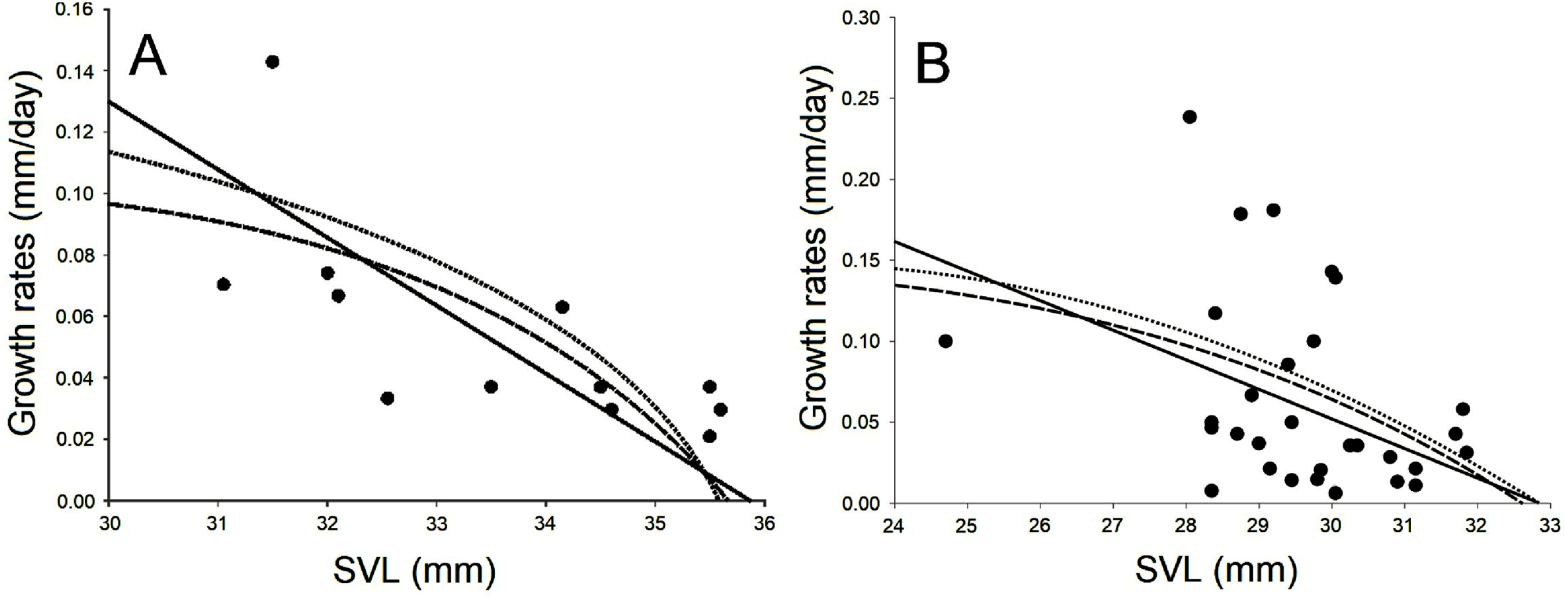
Observed growth rates based on body size in females and males of *Hyla eximia*. Observed growth rates (•) based on body size in females (A) and males (B) of *Hyla eximia*. The lines represent the expected relationship between the growth rates and the sizes of both sexes according to the Von Bertalanffy model (solid line), logistic-by-length model (broken line) and logistic-by-weight model (dotted line).

The GR for males and females corresponded to predictions from the logistic-by-weight and Von Bertalanffy models, respectively (Fig. 4; Table 4). In males, the selected model showed a higher value (*r* ± EE: 0.025 ± 0.008/day) than the selected model for females (*r* ± EE: 0.013 ± 0.004/day). Conversely, the value of the asymptotic growth parameter in males was lower (A_1_ ± EE: 32.76 ± 1.21) than that for females (A_1_ ± EE: 37.33 ± 1.42; Table 4), indicating that females reach larger body sizes (SVL) than males. Residuals of GR for females showed no relationship with environmental temperature (*r* = 0.24, *P* = 0.42) or temperature of microhabitat (*r* = 0.35, *P* = 0.24); in contrast, for males, both of these factors were negatively correlated with GR (environmental temperature: *r* = −0.39, *P* = 0.029; microhabitat temperature: *r* = −0.38, *P* = 0.038, respectively).

**Table 4:** Estimated parameters for males and females of *Hyla eximia*.

Models	MSR	*R*^2^	A_1_	*r*
Males (30)				
Von Bertalanffy	0.090	0.35	33.89 ± 2.22	0.015 ± 0.007
Logistic-by-length	0.089	0.36	33.16 ± 1.54	0.020 ± 0.008
Logistic-by-weight	0.089	0.37	32.76 ± 1.21	0.025 ± 0.008
Females (13)				
Von Bertalanffy	0.003	0.66	37.33 ± 1.42	0.013 ± 0.004
Logistic-by-length	0.007	0.65	37.04 ± 1.18	0.016 ± 0.004
Logistic-by-weight	0.007	0.64	36.8 ± 1.01	0.19 ± 0.004

**Notes:**

Estimated parameters for males and females of *Hyla eximia* obtained from tree models of body growth. Value in parentheses corresponds to the number of recaptured specimens of each sex. Means presented ± standard error.

MSR, mean square residual; *R*^2^, coefficient of determination; *r*, characteristic growth parameter; A_1_, asymptotic body size.

## Discussion

### Sexual dimorphism

Sexual dimorphism in anurans has been found in many morphological characteristics, such as body size (SVL), body shape, and color pattern (Hoffman & Blouin, 2000). In anuran species, sexual dimorphism of body size is female-biased, which is related to female fecundity (Salthe & Macham, 1974; Wells, 2007; Nali et al., 2014). A similar pattern was found in this population of *H. eximia*; females were larger in SVL and in other analyzed morphological characteristics. This pattern is similar to previous reports of congeners *H. euphorbiacea* (Luría-Manzano & Gutiérrez-Mayén, 2014), *H. chrysoscelis* (Ritke, Babb & Ritke, 1990), and *H. japonica* (Hirai & Matsui, 2000). This information is supported by the results of the SSD analysis in this study, which indicate that females exhibited larger morphological structures than males. Notably, body sizes in this study were larger than those reported for other populations of the same species (Cruz-Ruiz et al., 2015). Female-biased sexual dimorphism in *H. eximia* from our study population could be explained not only in terms of fecundity, but also GRs; the GRs of females is slower than that of males, allowing them to reach sexual maturity at a larger size. Alternatively, males should exhibit a high rate of growth to reach sexual maturity (at smaller SVL) before females to effectively compete with other males for access to calling sites, and so maximize their number of matings (Wells, 2007; Lode & Le Jaques, 2003). ANCOVAs showed that the increase in HL, HW, AL, FL, and FML with respect to SVL was greater in females than males. This result is consistent with the data obtained in an important study by Nali et al. (2014); they analyzed 616 species of frogs and found that the body dimensions of the females were significantly greater than that of the males, which in turn was related to fecundity (clutch size). According to Nali et al. (2014), size-dependent selection in females associated with fecundity and breeding patterns is an important mechanism driving SSD evolution in frogs.

### Phenology

Males emerged at the beginning of the first rain of the year (May), and both male calling and amplexus occurred in June and July during the rainy period (INEGI, 1992; Pavón & Meza-Sánchez, 2009). Calling behavior extended from June to September, a period in which we detected amplexus, egg laying, and larvae in various developmental stages (Duellman, 2001; Berns, 2013). This pattern suggests that females of *H. eximia* from this population may have more than one egg clutch during the reproductive season (Duellman, 2001).

The mean clutch size of this population (851.1 ± 106.9 eggs) was lower than that reported for *H. cinerea* (1,214 ± 528 eggs, *n* = 51; Gunzburger, 2006), but was similar to that of *H. gratiosa* (867 ± 331 eggs, *n* = 11; Gunzburger & Travis, 2007), and higher than that reported for *H. euphorbiacea* (774 ± 105 eggs, *n* = 12; Luría-Manzano & Gutiérrez-Mayén, 2014). Similarities in clutch size could be related to phylogenetic history (Haddad & Sawaya, 2000; Gómez-Mestre, Pyron & Wiens, 2012; Crump, 2015), but may also reflect similar responses to local environmental factors, such as precipitation, humidity, photoperiod, and/or solar radiation (Cruz-Ruiz et al., 2015; Lemos-Espinal et al., 2016).

On the other hand, male reproductive activity inferred from weight and testes volume has been very little studied in the genus *Hyla*, and mainly in *H. eximia* (Cruz-Ruiz et al., 2015). Luría-Manzano & Gutiérrez-Mayén (2014) reported a higher testicular volume for male *H. euphorbiacea* (105 ± 97.2 mm) than we found in males of our study population of *H. eximia* (20.11 ± 6.33 mm). This difference may be related to be the larger body size (SVL) of males in the former species as compared to *H. eximia*. The reproductive activity of male *H. eximia* is reflected in the variation of testis weight and volume among months, as the increase in these variables was related to the onset of the first rains (May–June), reaching peak values in June and decreasing from August to September. This pattern may be related not only to the environment but might reveal an underlying phylogenetic effect (Gómez-Mestre, Pyron & Wiens, 2012; Crump, 2015), as it is similar to that of other species of the genus, such as *H. arenicolor* (Woolrich-Piña et al., 2011), *H. euphorbiacea* (Luría-Manzano & Gutiérrez-Mayén, 2014), and *H. plicata* (Lemos-Espinal et al., 2016).

Larval development occurred during the period of highest precipitation (July and September), similar pattern to of *H. plicata* (Lemos-Espinal et al., 2016), but different from that of *H. arenicolor* (Davis & Smith, 1953) and *Exerodonta xera* (January–November; Canseco-Márquez, Gutiérrez-Mayén & Mendelson, 2003). In these latter species, the period of reproductive activity is longer (January–October), and therefore, the period of larval development is longer (i.e., more variation in the number of development stages observed at any single point in the reproductive season).

### Growth rate

The logistic-by-weight model best described the GRs of males, whereas the Von Bertalanffy model best described growth in females. Thus, GRs analysis showed that males grew and reached a body size associated with sexual maturity faster than females but attained a smaller overall body size than females. This pattern has been found in other species of the genus *Hyla* in general (Shine, 1979), including species in the *eximia* group specifically (Ritke, Babb & Ritke, 1990; Hirai & Matsui, 2000).

Correlations between the residuals of GRs and environmental and microhabitat temperature of adult female *H. eximia* were not significant; however, as tadpoles, their growth might depend on water temperature and availability of aquatic food (freshwater algae; Dmitriew, 2011). McMillen & Robinson (2005) pointed out that accelerated growth in tadpoles is a strategy to avoid predation, which gradually decreases until they become subadults or adults. However, quality and quantity of food could significantly influence the growth of adult female *H. eximia*, as occurs in other groups of vertebrates such as lizards (Stamps & Tanaka, 1981; Dmitriew, 2011). We observed a negative and significant relationship among residuals of the GRs with both environmental and microhabitat temperature in males. This pattern was similar to those reported for males of the European frog *Pelophylax lessonae* (Orizaola & Laurila, 2015). According to Orizaola & Laurila (2015), this relationship reflects accelerated growth in the first phase of the transition of metamorphosis (larval to juveniles), followed by a reduction in GRs when individuals reach adulthood and prepare to reproduce, thus representing trade-offs between reproduction and growth (Stearns, 1992; Dmitriew, 2011).

### Diet

Males consumed fewer prey than females; this might reflect the fact that males are smaller in body size, and therefore their stomachs as well. This pattern is similar to that of other members of the genus, such as *H. euphorbiacea* (Luría-Manzano & Gutiérrez-Mayén, 2014), as well as other anuran species (Paton, Stevens & Longo, 2000). Another possible explanation for the number of prey eaten by males is related to reproductive behavior (Wells, 2007); during the reproductive season, males invest more time and energy in looking for optimal sites to call and amplexus mates (Duellman & Trueb, 1986; Luría-Manzano & Gutiérrez-Mayén, 2014) than to forage, and a similar pattern has been observed in *H. cinerea* (Leavitt & Fitzgerald, 2009).

Differences in the reproductive behavior and body size of males and females are related to the type and number of consumed prey in each sex (Cogalniceanu, Palmer & Ciubuc, 2000; Luría-Manzano & Ramírez-Bautista, 2017), and each sex is therefore able to adopt different strategies for foraging (Toft, 1980). Males of *H. eximia* exhibit sit-and-wait foraging (Wells, 2007), whereas females could potentially continue to feed when moving during amplexus or searching for egg laying sites (Wells, 2007; Luría-Manzano & Gutiérrez-Mayén, 2014). These behaviors permit a different diet composition between the sexes, such as occurs in other anuran species (Toft, 1980; Parmelee, 1999; Leavitt & Fitzgerald, 2009; Luría-Manzano & Gutiérrez-Mayén, 2014).

Despite recording a greater number of categories of prey consumed by females (nine) than in males (seven), the Coleoptera and Diptera were the most important prey items to both sexes, both in abundance and in volume. Likewise, plant material consumed by females and males was high, suggesting this type of food is an intentional part of the diet of individuals of this population, and not simply an inadvertent by-product of predation. Plant material consumed by anurans has been reported in other frogs, such as *Incilius valliceps* (Gelover, Altamirano & Soriano, 2001) and *Lithobates vaillanti* (Ramírez-Bautista & Lemos-Espinal, 2004). Interpretations for an intentional consumption of plant matter include that this type of diet acts as an important source of water, as well as facilitates the elimination of intestinal parasites, or helps with the fermentation of food (Anderson, Haukos & Anderson, 1999; Mendoza-Estrada, Lara-López & Castro-Franco, 2008).

The dominance of a small number of prey categories in the diet of both sexes may indicate similar preferences in the use of resources available in the environment (Leavitt & Fitzgerald, 2009), but the different prey types consumed by females generates low values of dietary overlap. Consequently, to better evaluate the feeding niches of each sex it will be necessary to carry out studies that assess the use and availability of food resources in the environment, as well as dimensions of home range of individual frogs (Cruz-Ruiz et al., 2015; Luría-Manzano & Ramírez-Bautista, 2017).

## Conclusion

Our results provide important advances in the knowledge of the ecology of this species. This study revealed differences in GRs as compared to other populations of the same species (Cruz-Ruiz et al., 2015). In addition, data on life history, such as clutch size, growth, phenology, and diet, help describe the behavior and natural history of this population of *H. eximia*, in turn serving as a good model for other studies in different populations of this species and others.

## Supplemental Information

10.7717/peerj.5897/supp-1 Supplemental Information 1 Dryophytes eximius records for this study.

10.7717/peerj.5897/supp-2 Supplemental Information 2 Measurements and age classes.

## Additional Information and Declarations

### Field Study Permissions

The following information was supplied relating to field study approvals (i.e., approving body and any reference numbers): Field work was conducted according to the Guidelines for the Care and Use of Lower Vertebrates (2004), and the national Mexican laws CT-CERN-001-91 (DOF, 1991) and NOM-PA-CRN-001/93 (DOF, 1993). Specimens were collected under scientific permit SGPA/DGVS/01902/11 provided by SEMARNAT.

### Data Availability

The following information was supplied regarding data availability: The raw data are available as Supplemental Files.
